# Biotransformation of Androstenedione by Filamentous Fungi Isolated from Cultural Heritage Sites in the State Tretyakov Gallery

**DOI:** 10.3390/biology11060883

**Published:** 2022-06-08

**Authors:** Alexander A. Zhgun, Mark P. Potapov, Darya A. Avdanina, Natalya V. Karpova, Vera V. Yaderets, Vakhtang V. Dzhavakhiya, Dmitry A. Kardonsky

**Affiliations:** 1 Research Center of Biotechnology, Russian Academy of Sciences, 119071 Moscow, Russia; mrk9804@gmail.com (M.P.P.); d.avdanina@gmail.com (D.A.A.); ashatanr@mail.ru (N.V.K.); verayaderetz@yandex.ru (V.V.Y.); dzhavakhiya@biengi.ac.ru (V.V.D.); 2 Federal Research and Clinical Center of Physical-Chemical Medicine of Federal Medical Biological Agency, 119435 Moscow, Russia; freddy1178@yandex.ru

**Keywords:** steroid biotransformation, filamentous fungi, biodeterioration of tempera painting, cholesterol, androst-4-en-3,17-dione

## Abstract

**Simple Summary:**

Microorganisms are able to grow on substrates of the most diverse nature. One of the most practical habitats, in terms of cultural heritage conservation, is fine art objects such as tempera or oil paintings on canvas. Since tempera paints are produced on the basis of egg yolk, which is one of the richest sources of cholesterol in nature (up to 2% of dry weight), and in the process of aging of tempera materials, changes in cholesterol do not affect the core structure of the steroid nucleus, the group of fungi that we have isolated are tempera painting destructors is seen as a promising object for screening for their possible steroid-transforming activities. In this regard, the purpose of our work was to determine the ability to transform pharmaceutically significant steroids with dominant fungi-destructors of tempera paintings, previously isolated in the State Tretyakov Gallery. Consequently, we have demonstrated for the first time that fungi-destructors of tempera paintings have steroid-transforming activity and are promising microorganisms for screening for biotechnologically significant transformations of steroids with further industrial use.

**Abstract:**

The transformation of steroids by microorganisms is widely used in medical biotechnology. A huge group of filamentous fungi is one of the most promising taxa for screening new biocatalytic reactions in order to obtain pharmaceutically significant steroids. In this work, we screened 10 filamentous fungi-destructors of egg tempera for the ability to biotransform androst-4-en-3,17-dione (AD) during cultivation in a liquid nutrient medium or in a buffer solution. These taxonomically unrelated strains, belonging to the classes *Eurotiomycetes*, *Dothideomycetes* and *Sordariomycetes*, are dominant representatives of the microbiome from halls where works of tempera painting are stored in the State Tretyakov Gallery (STG, Moscow, Russia). Since the binder of tempera paints, egg yolk, contains about 2% cholesterol, these degrading fungi appear to be a promising group for screening for steroid converting activity. It turned out that all the studied fungi-destructors are able to transform AD. Some strains showed transformation efficiency close to the industrial strain *Curvularia lunata* RNCIM F-981. In total, 33 steroids formed during the transformation of AD were characterized, for 19 of them the structure was established by gas chromatography/mass spectrometry analysis. In this work, we have shown for the first time that fungi-destructors of tempera paintings can efficiently transform steroids.

## 1. Introduction

The annual world production of the steroid market is more than USD 10 billion and is at the forefront of the pharmaceutical industry [1]. Currently, about 300 steroid drugs are known, meaning that steroids production occupies the second position in the pharmaceutical market after the production of antibiotics [2]. The first pharmaceutically significant steroid drugs were obtained in the 1950s as a result of multi-stage chemical syntheses [3]. Later, it turned out that the number of stages for obtaining the target steroid can be significantly reduced using biotransformation by microorganisms [1,4]. In many cases, the benefits of steroid biotransformation are due to single step regio- and/or stereospecific reactions that are catalyzed by specially «sharpened» enzymes, and which are not always available for chemical synthesis [1,5,6]. The steroid core structure is also sensitive to cleavage by numerous chemicals, and the use of biotransformation can sometimes solve a particular synthesis problem [5,7]. In addition, steroid biotransformation is a more environmentally friendly process compared to many stages of chemical synthesis [1,5]. The ability to transform pharmaceutically significant steroids has been found in various groups of microorganisms [2,8]. Based on these studies, improved strains of microorganisms have been obtained for highly efficient transformations of various steroids [1]. Among them are improved strains of filamentous fungi (also called molds or moldy fungus), for example, *Curvularia lunata* RNCIM F-981 for 14α-hydroxylation of androst-4-ene-3,17-dione (AD, androstenedione) [9]. Currently, more then 70,000 filamentous fungi are known [10], and some of them contain unusual or unique reactions for secondary metabolites biosynthesis [11,12,13] and biotransformation of steroids [14,15,16]. For example, the individual species of filamentous fungi contain 50–100 or more cytochromes P450 (CYP450) [17], some of them (10–30% of the total) catalyze oxidative hydroxylation reactions and other chemical modification of steroids [15,18]. However, for the overwhelming majority of molds, the “capacity” of steroid biotransformation has not been characterized [2,19]. In this regard, among various groups of filamentous fungi, active screening for the ability to transform pharmaceutically significant steroids is being carried out [20,21,22,23].

Previously, we collected 106 microbiological samples from exhibits of tempera paintings, related to cultural heritage, and surfaces of the Paintings of Ancient Rus Halls in the main building of the State Tretyakov Gallery (STG, Moscow, Russia) [24]. We also characterized uncultivated and cultured microorganisms using metagenomic sequencing and showed that dominant representatives of bacterial and fungal microbiomes can be cultivated on standard microbiological media (project accession no: https://www.ncbi.nlm.nih.gov/sra/PRJNA606688/ (accessed on 14 February 2020). After inoculation of isolated cultures on the created mock layers (with organic materials used in tempera painting) the main potential biodestructors of the studied objects of cultural heritage were identified [24]. They turned out to be 10 filamentous fungi that destroy tempera paintings. In this regard, this group of fungi was characterized in detail in terms of macromorphology and micromorphology, and new effective antiseptics were selected against them, which can be used during planned or emergency conservation [25,26,27].

The main binder for pigment paints in tempera painting is chicken egg yolk [24]. Liquid egg yolk contains about 1% cholesterol, which is one of the highest in nature; when tempera paints dry, the cholesterol content increases to 2% or more [28,29]. It was shown that in the process of the aging of works of tempera painting, modifications of cholesterol appear, but they are not associated with the degradation of steroid rings; the core structure of the steroid nucleus is preserved [30]. In this regard, fungi destructors of tempera paintings can be a promising object for screening the biotransformation ability of pharmaceutically significant steroids.

Based on this, the goal of our work was to study the biotransformation of AD (steroid hormone with weak androgenic action and one of the most important intermediates in the biotransformation of steroids) by filamentous fungi, capable of destroying tempera painting materials at cultural heritage sites of the State Tretyakov Gallery.

## 2. Materials and Methods

### 2.1. Materials

Androst-4-ene-3,17-dione (AD) from Sigma-Aldrich (St. Louis, MO, USA) was used as initial substrate and as standard. Androsta-1,4-diene-3,17-dione (ADD) and testosterone (TS) from Sigma-Aldrich (USA) were used as standards. 7α-OH-testosterone, 9α-OH-androst-4-ene-3,17-dione, 11α-OH-androst-4-ene-3,17-dione, 14α-OH-androst-4-ene-3,17-dione, and 14α-OH-Androsta-1,4-diene-3,17-dione were taken from the laboratory collection and were used as standards.

### 2.2. Microorganism Strains Used in the Work

The following strains of filamentous fungi were used to determine the activity of steroid biotransformation: 10 micromycete strains, isolated previously from [24], *Aspergillus versicolor* STG-25G (SRX7729174; MK260015.1), *Ulocladium* sp. AAZ-2020a STG-36 (MW590700.1; SRX7729176), *Cladosporium halotolerans* STG-52B (SRX7729178; MK258720.1), *Aspergillus creber* STG-57 (SRX7729151; MK266993.1), *Aspergillus versicolor* STG-86 (SRX7729182; MK262781.1), *Aspergillus creber* STG-93W (SRX7729186; MW575292.1), *Cladosporium parahalotolerans* STG-93B (SRX7729188; MK262909.1), *Simplicillium lamellicola* STG-96 (SRX7729192; MK262921.1), *Microascus paisii* STG-103 (SRX7729190; MW591474.1), and *Aspergillus protuberus* STG-106 (SRX7729192; MK268342.1) and the industrial strain *Curvularia lunata* RNCIM F-981 [9].

### 2.3. Cultivation of Fungal Strains

The filamentous fungi, isolated from STG, were cultivated on slant agarized Czapek-Dox (CDA) medium (30 g/L sucrose, 2 g/L NaNO_3_, 1 g/L K_2_HPO_4_, 0.5 g/L MgSO_4_ × 7H_2_O, 0.5 g/L KCl, 0.01 g/L FeSO_4_ × 7H_2_O, 20 g/L agar, pH 7.0–7.4) at 24–26 °C. *C. lunata* RNCIM F-981 was cultivated on slant agarized T2D medium (4 g/L yeast extract, 10 g/L malt extract, 4 g/L glucose, 30 g/L agar, pH 6.2–6.9) at 28 °C.

### 2.4. Preparation of Fungal Strains for Steroid Transformation

After 10–15 days of cultivation on agar slant, fungal cultures were collected with 10 mL of 0.9% NaCl solution; 5 mL of this fungal cell mixture was inoculated into 40 mL of liquid seed (SE) medium. For strains from STG, medium F (30 g/L sucrose, 5 g/L yeast extract, 2 g/L NaNO_3_, 3 g/L (NH_4_)H_2_PO_4_, 0.5 g/L KCl, 1 g/L MgSO_4_ × 7H_2_O, pH 5.5–6.0) was used as SE medium; for *C. lunata* RNCIM F-981, medium F or medium H (30 g/L sucrose, 7 g/L yeast autolysate, 2 g/L NaNO_3_, 1 g/L KH_2_PO_4_, 3 g/L (NH_4_)H_2_PO_4_, 0.5 g/L KCl, 0.5 g/L MgSO_4_ × 7H_2_O, pH 6.0–6.1) were used as SE medium. Seed cultures were grown for 72 h on a rotary shaker CERTOMAT ^®^BS-1 (Sartorius, Germany) at 220–240 rpm in 250 mL Erlenmeyer flasks at 26 °C.

### 2.5. Steroid Biotransformation

The cultures of filamentous fungi pre-grown on SE medium were then used to study their ability to transform steroids. For this, the cells were either transferred to a liquid nutrient medium (TM1, transformation method 1) or into a potassium phosphate buffer (TM2, transformation method 2). Stock steroid substrate (AD) suspension was prepared each time immediately before application for biotransformation. The AD was ground in a mortar and pestle to form particles with a size of approximately 15–20 μm and mixed with Tween 80 (2.5% solution in water) to obtain a stock AD suspension with a concentration of 40 g/L.

In the case of TM1, 10 mL of culture from the SE medium was inoculated into 40 mL of liquid defined (DE) medium for steroid transformation. For strains from STG, medium F was used as DE medium; for *C. lunata* RNCIM F-981, medium F or medium N (30 g/L sucrose, 5 g/L peptone, 5 g/L yeast extract, 10 g/L soybean meal, 2 g/L KH_2_PO_4_, 0.5 g/L MgSO_4_ × 7H_2_O, pH 6.2) were used as DE medium. Liquid cultures were grown for 24 h at 220–240 rpm in 250 mL Erlenmeyer flasks at 26 °C. Then, aliquot of stock AD suspension was added to a final concentration of 1 g/L. Bioconversion was carried out on a rotary shaker (220 rpm) at 26 °C for a maximum of 72 h and monitored daily by TLC and GC/MS as described below. The data recorded were measured in triplicate and repeated at least twice.

In the case of TM2, 10 mL of culture from the SE medium was inoculated into 40 mL of the same type of SE medium (medium F—for strains from STG, medium H—for *C. lunata* RNCIM F-981); grown for 24 h at 220–240 rpm in 250 mL Erlenmeyer flasks at 26 °C. Then, the mycelium was separated from the culture liquid by filtration and collected on a Flag Grid filter with a pore diameter of 15–20 µm (EuroFlag, Russia, Moscow), washed with 10 volumes of 150 mM potassium phosphate buffer (PPB, pH 6.0). Next, 4 g of the mycelium wet biomass were inoculated into 40 mL of 150 mM potassium phosphate buffer (pH 6.0). Then, aliquot of stock AD suspension was added to a final concentration of 1 g/L. Bioconversion was carried out on a rotary shaker (220 rpm) at 26 °C for a maximum of 72 h and monitored daily by TLC and GC/MS as described below. The data recorded were measured in triplicate and repeated at least twice.

### 2.6. Sample Preparation

Aliquots of 2 mL of the cultivation broth were taken after 24, 48, and 72 h incubation of fungal strains with steroid substrates in DE medium or in PPB. The steroid compounds were extracted with two volumes of ethyl acetate (EtOAc), 2 h, 20–25 °C; then stored at 4 °C.

### 2.7. Thin Layer Chromatography (TLC)

To determine the biotransformation products, 40 μL of EtOAc extracts were applied to TLC plates with Sorbfil UV 254 (Sorbfil, Russia), developed in an acetone–chloroform mixture (7:3, *v*/*v*), and visualized under UV light (254 nm) on Desaga HP-UVIS (Desaga, Germany). A color reaction was also used for visualization; for this, a 1% solution of vanillin in a 10% aqueous solution of HClO_4_ was applied to a chromatographic plate and developed at a temperature of 70–80 °C. The samples of cultivation broth were monitored daily.

### 2.8. Gas Chromatography/Mass Spectrometry (GC/MS)

Steroids were determined on the GCMS-QP2010 Ultra Gas Chromatograph Mass Spectrometer (Shimadzu, Japan, Kyoto) from 1 μL of the samples. The column J&W DB-5 ms Ultra Inert GC Column, 30 m, 0.25 mm, 0.25 µm, 7-inch cage (Agilent Technologies Inc., Santa Clara, CA, USA) was used. Chromatography conditions: column temperature 100 °C, heating at a rate of 20 °C/min to 280 °C, holding for 20 min; carrier gas—helium, injecto—split (1:10), carrier gas flow rate—1 mL/min, quadrupole analyzer temperature—150 °C. The temperature of the ion source was 230 °C, the time of switching on the cathodes and the analyzer (“solvent delay”) was 3.51 min after the injection of the sample, and the interval of the scanned masses was 50–750 *m*/*z*. The detector operating mode was set according to the standard “Autotune” program. The mass spectrum libraries NIST [31] and REAXYS (http://www.reaxys.com accessed on 21 March 2022) were used to identify steroid compounds based on the obtained mass spectra.

## 3. Results

### 3.1. Transformation of AD by Fungal Strains

The ability to transform AD was studied for 10 filamentous fungi, previously isolated from the exhibits and halls of the STG and showed the biodegradation of paints and varnishes used in tempera paintings [24]. Five species of fungi were from the family *Aspergillaceae* (*Aspergillus versicolor* STG-25G, *A. creber* STG-57, A. versicolor STG-86, A. creber STG-93W, *A. protuberus* STG-106), two species were from the family *Cladosporiacea* (*Cladosporium halotolerans* STG-52B, *C. parahalotolerans* STG-93B) and one representative each from the families *Pleosporaceae* (*Ulocladium* sp. AAZ-2020a STG-36), *Cordycipitaceae* (*Simplicilium lamellicola* STG-96) and *Microascaceae* (*Microascus paisii* STG-103). An industrial strain of filamentous fungus *Curvularia lunata* RNCIM F-981 (from the family *Pleosporaceae*) [9] was used as a control.

**Figure 1:**
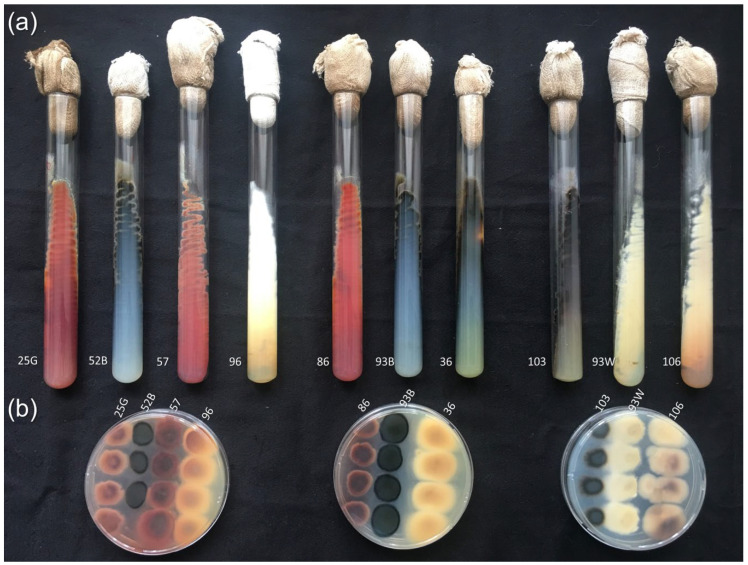
Cultivation of fungi-destructors of tempera paintings, isolated in the State Tretyakov Gallery (STG, Moscow, Russia) on Czapek-Dox agar (CDA) medium, 25 °C, 15 days. (**a**) Slant CDA medium; (**b**) CDA medium on Petri dishes. Fungal strains, from left to right: STG-25G, STG-52B, STG-57, STG-96, STG-86, STG-93B, STG-36, STG-103, STG-93W, STG-106.

The strains were preliminarily cultured on Czapek-Dox (CDA) agar medium for 10–15 days (Figure 1).

**Figure 2:**
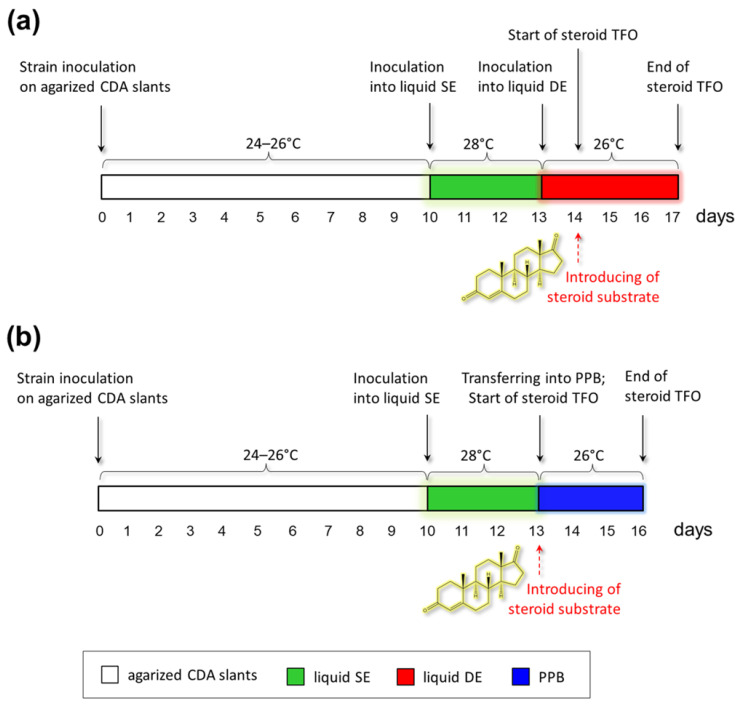
Timelines schemes of steroid conversion by fungi-destructors of tempera paintings: (**a**) steroid transformation into liquid defined (DE) medium; (**b**) steroid transformation into potassium phosphate buffer (PPB). CDA—Czapek-Dox agar medium; SE—liquid seed (SE) medium; TFO—transformation. The red dashed arrow shows the time of steroid substrate introduction.

**Figure 3:**
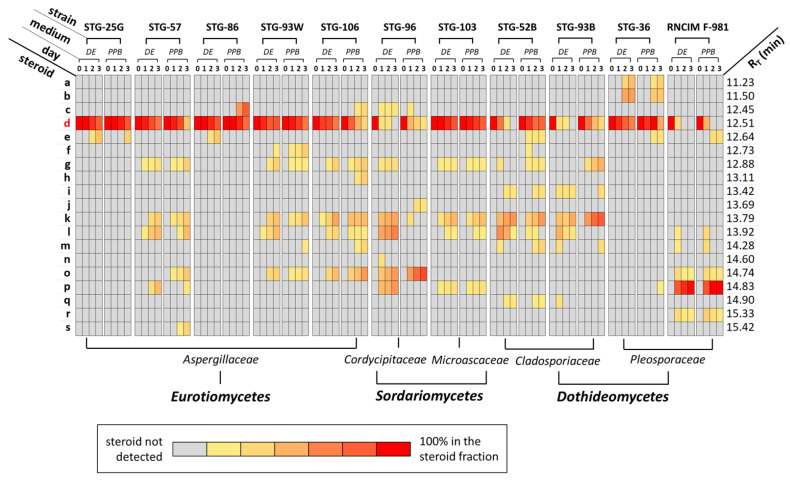
The dynamic of androst-4-ene-3,17-dione (**d**) transformation by filamentous fungi-destructors of tempera painting materials, isolated in the State Tretyakov Gallery, Moscow, Russia (STG-25G, STG-57, STG-86, STG-93W, STG-106, STG-96, STG-103, STG-52B, STG-93B, STG-36), and by the industrial strain *Curvularia lunata* RNCIM F-981 (control). Data on 0, 1, 2, and 3 days after the addition of 1 g/L substrate (**d**) is based on gas chromatography/mass spectrometry analysis. DE—liquid defined medium, PPB—potassium phosphate buffer. Substrate (**d**) in bold red; all other compounds (**a**–**c**, **e**–**s**) are steroid conversion products and are not colored (black, bold). R_T_—retention time.

Then, the fungal cells were transferred to a liquid seed (SE) medium for growing biomass during 48–72 h (Figure 2). After that, either (i) an aliquot of the culture liquid was inoculated onto the liquid defined (DE) medium for transformation, grown for 24 h, then the steroid substrate (AD) was added and transformation was carried out; or (ii) the obtained biomass was collected on a filter, washed from the culture liquid with a buffer solution (150 mM potassium phosphate buffer, PPB) and transferred to PPB supplemented with a steroid substrate for the transformation. At the start of steroid transformation and every day after (Figure 2), aliquots of samples were taken for analysis by thin layer chromatography (TLC) and gas chromatography/mass spectrometry (GC/MS). The preliminary content of steroids in the samples was estimated by TLC, and then these data were verified by GS-MS analysis. The GC/MS values and the structures of the initial steroid substrate and its bioconversion products resulting from transformation both in the DE medium and in PPB are presented in Table 1. Encoding for the substrate (AD) and bioconversion products, from **a** to **s**, corresponding to their retention time (RT) in GC/MS analysis is also introduced (Table 1). The dynamics of transformation of compound **d** by fungi-destructors of tempera painting materials and the industrial strain *C. lunata* RNCIM F-981 (control) are shown in Figure 3. Compounds and their relative amount determined in samples after 0, 1, 2 and 3 days of transformation in DE medium and in PPB by the GC/MS method. At the start of transformation (day 0, time immediately after AD addition), only this compound **d** was found in the samples; then, during the transformation, its amount decreased, and the products of steroid transformation were detected (Figure 3). For example, for strain STG-36, the AD substrate detected at the beginning of the process (Supplementary Materials, Figure S1) after 2 days of transformation was partially converted to steroid compounds **a** and **b** (Supplementary Materials, Figure S2). Steroid transforming activity was found for all classes of fungi studied: *Eurotiomycetes* (e.g., STG-57, Supplementary Materials, Figure S3), *Sordariomycetes* (e.g., STG-96, Supplementary Materials, Figure S4) and *Dothideomycetes* (e.g., STG-93B, Supplementary Materials, Figure S5).

#### 3.1.1. Transformation of AD by Fungal Strains during Cultivation on DE Medium

For the primary screening for the ability of fungi-destructors of tempera paintings to transform steroids, we used as a basis the previously developed scheme for the transformation of AD (**d**) by the strain *C. lunata* RNCIM F-981 [32]. A substrate loading of 1 g/L and a previously developed biotransformation scheme using F medium as both SE and DE mediums was used (transfer F medium to F medium) [20]. In the control experiment, we also transformed substance **d** (1 g/L) with *C. lunata* RNCIM F-981, using H medium as SE medium and N medium as DE medium (transfer H medium to N medium). Under such conditions, previously optimized for this industrial strain [32], we observed a characteristic and specific transformation of the compound **d** into **p**. When applying the transfer scheme from medium F to medium F for the transformation of **d** with *C. lunata* RNCIM F-981, the major substance was **p**, but the appearance of **l** and **m** (after the first day), as well as **o** and **r** (throughout the entire period) was also observed (Figure 3). It is also important to note that, under these conditions, *C. lunata* RNCIM F-981 completely metabolized substrate **d**, 2 days after its addition. This allowed us to use the transformation activity of *C. lunata* RNCIM F-981 on medium F as a relative control to assess the activity of STG strains.

It turned out that all STG strains are able to transform AD on DE medium (Figure 3). Strains STG-25G and STG-86 showed similar transformational activity; they specifically accumulated testosterone (compound **e**) after 2 days (Supplementary Materials, Figure S6). During the transformation of AD with strain STG-57, compounds **g** and **l** were detected (after 1 day), then additional compounds **l** and **k** were detected (after 2 days, Supplementary Materials, Figure S3). During the transformation with STG-93W, compound **l** was detected (after 1 day), then **g**, **k**, and **o** were additionally detected (after 2 days), then **f** was additionally detected (after 3 days). During the transformation of AD with STG-106, compound **k** was detected (after 1 day), then compounds **g**, **l**, and **o** were additionally detected (after 2 days). After 3 days of transformation, all Aspergillus samples partially retained the original AD substrate. One of the best activities under these transformation conditions was shown by strain STG-96. After 2 days of transformation, only traces of the original AD substrate (Supplementary Materials, Figure S4) were detected; AD completely disappeared after 3 days of transformation (Figure 3). After 1 day of transformation, **c**, **g**, **k**, **l**, **n**, **o** and **p** compounds were found in the sample. After 2 days of transformation, **n** was not detected, all other compounds were present in the samples until the end of transformation (up to 3 days). When transforming AD with strain STG-103, compounds **g**, **k** and **p** were detected (after 1 day); then compound l was additionally detected (after 2 days). *Cladospores* (STG-52B and STG-93B), as well as strain STG-96, completely metabolized AD after 3 days of incubation. In the process of transformation, products **g**, **j**, **k**, **l**, **m** and **q** were detected at different stages. The main compound after 3 days was **k**. Strain STG-36 after 2 days produced unique (for the studied group of fungi) compounds **a** and **b** (Supplementary Materials, Figure S2).

#### 3.1.2. Transformation of AD by Fungal Strains in PPB

In order to more extensively reveal the transformational potential of fungi-destructors of egg tempera, we also studied the bioconversion of **d** in PPB. For this, we used the experimental design described earlier [20], when the biomass grown on the SE medium was transferred to PPB supplemented with **d** (1 g/L). Such a change in conditions made it possible for most strains to detect both the formation of novel steroids and altered concentration dynamics for previously detected steroids (Figure 3). Thus, for strain STG-25G, compound **e** was detected in PPB one day later than during steroid transformation in SE medium. Transformation with strain STG-57 in PPB revealed additional substances **o** and **s**; the changed dynamics were for substances **k**, **l** and **p**. For strain STG-86, on the second day, substance **c** appeared and **e** disappeared. For strain STG-93W, steroid **m** appeared on the third day and steroid **l** disappeared; the changed dynamics was for substances **f**, **g**, **k** and **o**. For strain STG-106, significant changes occurred during transformation in PPB, substances **c**, **h**, and **m** appeared; the changed dynamics were for **g**, **l**, and **o**. For the STG-96 strain, changes occurred not only at the level of steroid products, but also in the consumption of the initial steroid substrate (Figure 3). Substance **d** was completely consumed in SE medium during the first day of transformation, while in PPB **d** was detected during all 3 days. In contrast, no significant differences were found for the *M. paisii* STG-103 under both transformation conditions. *Cladosporium* strains (STG-52B and STG-93B) showed very similar dynamics of substrate consumption. For both strains, substrate **d** was completely consumed on DE medium (after 2 days) and was not completely consumed in PPB during the entire observation period. In addition, for STG-52B in PPB compounds **e** and **f** were specifically detected; the changed dynamics were for **g**, **l** and **m**. For STG-93B **g** appeared, substances **l** and **q** disappeared; the changed dynamics were for **i** and **m**. For strain STG-93W steroids **e** end **p** appeared during transformation in PPB; no other changes were found.

### 3.2. Transformation of AD by Various Systematic Groups of Mold Fungi

To characterize the pathways of AD transformation by the filamentous fungi studied in our work and for comparison with the transformation activity described in the literature, we proposed transformation schemes for certain classes of mold fungi (*Eurotiomycetes*, *Dothideomycetes*, and *Sordariomycetes*) (Table 2).

All of these fungi belong to the same subdivision *Pezizomycotina*, the largest of the Ascomycota (with over 30,000 described species). *Pezizomycotina* is seen as one of the most promising systematic fungal groups for screening for steroid transformation activity due to its rich metabolic potential. In particular, it has one of the largest P450omes (CYP450 genes) [44,45], which can potentially serve as a source for the introduction of hydroxyl groups and other chemical modifications into various parts of the steroid core [15,18]. In this regard, we made an attempt to systematize the data described in the literature and obtained in our work on the transformation of AD by these groups of fungi.

#### 3.2.1. Transformation of AD by Representatives of *Eurotiomycetes*

**Figure 4:**
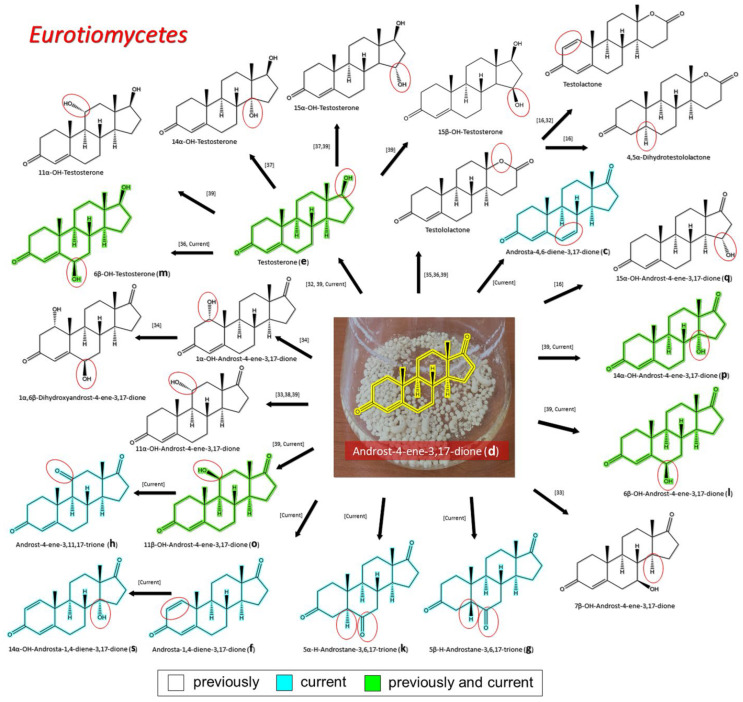
The transformation of androst-4-ene-3,17-dione (**d**) by *Eurotiomycetes* (family *Aspegillaceae*). Structural formula of the substrate (**d**) is drawn in yellow and placed on the image of the flask with defined (DE) medium after 48 h of transformation for by *Aspergillus versicolor* STG-86. Structural formulas of steroids detected during the transformation are drawn: in green for compounds found both in current and in other studies; in black for compounds found in other studies, but not found in current work; and in blue for compounds first discovered in current study. Next to the arrows are the literary sources, where modifications were described; modifications circled in red ovals.

The dominant representatives of *Eurotiomycetes* in the microbiome of the Old Russian painting halls of the State Tretyakov Gallery were strains from the *Aspergillaceae* family. It turned out that all the studied strains were able to transform AD, with different efficiency and specificity (Figure 3). Together with the current study, 23 possible steroid products have been described in the literature following the *Aspergillaceae* family steroid transformation of AD [16,33,34,35,36,37,38,39,40] (Table 2, Figure 4). Of the steroid products found in our study, 21.7% (5 out of 23) had been previously described: TS (**e**), 6β–OH–AD (**l**), 6β–OH–TS, (**m**), 11β–OH–AD (**o**), and 14α–OH–AD (**p**). Out of the steroidal products of AD transformation, 26% (6 out of 23) are described for the first time in our work: Androst-4,6-diene-3,17-dione (**c**), ADD (**f**), 5β–H–Androstane-3,6,17-trione (**g**), Androstane-3,11,17-trione (**h**), 5α–H–Androstane-3,6,17-trione (**k**), and 14α–OH–ADD (**s**).

#### 3.2.2. Transformation of AD by Representatives of *Dothideomycetes*

**Figure 5:**
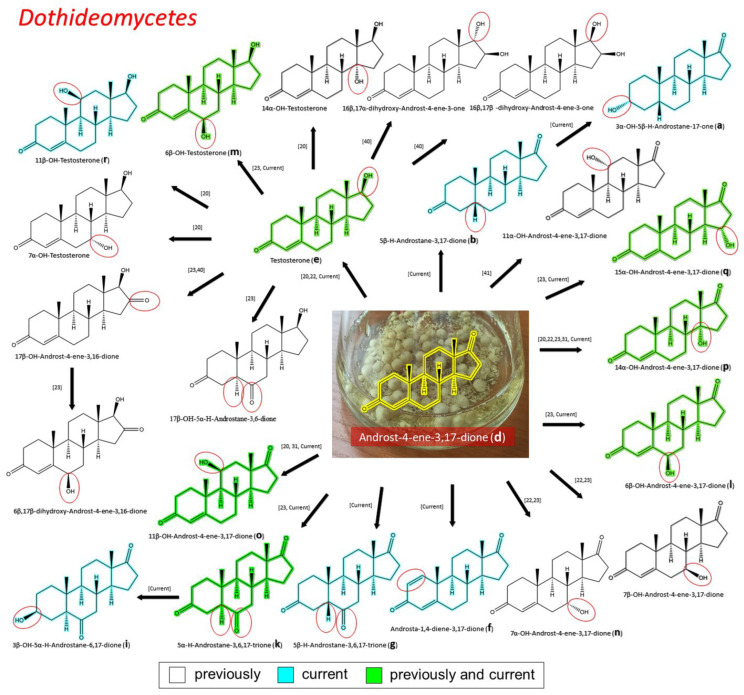
The transformation of androst-4-ene-3,17-dione (**d**) by *Dothideomycetes*. Structural formula of the substrate (**d**) is drawn in yellow and placed on the image of the flask with defined (DE) medium after 48 h of transformation for by *Ulocladium* sp. AAZ-2020a STG-36. Structural formulas of steroids detected during the transformation are drawn: in green for compounds found both in current and in other studies; in black for compounds found in other studies, but not found in current work; and in blue for compounds first discovered in the current study. Next to the arrows are the literary sources, where modifications were described; modifications circled in red ovals.

The dominant representatives of *Dothideomycetes* among the fungi-destructors of tempera paintings from STG were strains from the *Cladosporiaceae* and *Pleosporaceae* families. All the studied strains were able to transform AD, with different efficiency and specificity (Figure 3). For the *Pleosporaceae* family, we also performed AD transformation of the previously characterized *C. lunata* RNCIM F-981 control strain [20,32]. Under the studied conditions of AD transformation by the *C. lunata* RNCIM F-981 strain, we found the previously described major transformation product 14α-OH-AD (**p**) and by-products: 11β-OH AD (**o**) 11β-OH-TS (**r**) (Table 2). In addition, for the first time, we found compounds 6β–OH–AD (**l**) and 6β–OH–TS (**m**) for this strain, which indicates the presence of a previously undiscovered 6β-hydroxylating activity in this strain. Together with the current study, 23 possible steroid products have been described in the literature after steroid transformation of AD by fungal strains from the *Cladosporiaceae* and *Pleosporaceae* families [20,22,23,32,41] (Table 2, Figure 5). If we take into account the control strain *C. lunata* RNCIM F-981, then 30.4% (7 out of 23) of the steroid products found in our study were previously described (**e**, **k**, **l**, **m**, **o**, **p**, and **q**) and 26% (6 out of 23) of steroidal products are described for the first time (**a**, **b**, **f**, **g**, **i**, and **r**) (Figure 5). If we take into account only the fungi-destructors of tempera paintings, then 26% (6 out of 23) of the steroid products found in our study were previously described (**e**, **k**, **l**, **m**, **p**, and **q**) and 21,7% (5 out of 23) of steroidal products are described for the first time (**a**, **b**, **f**, **g**, and **i**), since compounds **o** and **r** were found only in the control strain *C. lunata* (Table 2). If we compare the features of transformation within the families of STG strains, then for *Dothideomycetes* 30.8% (4 out of 13) of the steroid products found in our study were previously described (**e**, **k**, **l**, and **q**) and 30.8% (4 out of 13) of steroidal products are described for the first time (**f**, **g**, **i**, and **m**) (Table 2). For *Pleosporaceae* (strain *Ulocladium* sp. AAZ-2020a STG-36), 20% (2 out of 10) of the steroid products found in our study were previously described (**e** and **p**) and 20% (2 out of 10) of steroidal products are described for the first time (**a** and **b**) (Table 2).

#### 3.2.3. Transformation of AD by Representatives of *Sordariomycetes*

**Figure 6:**
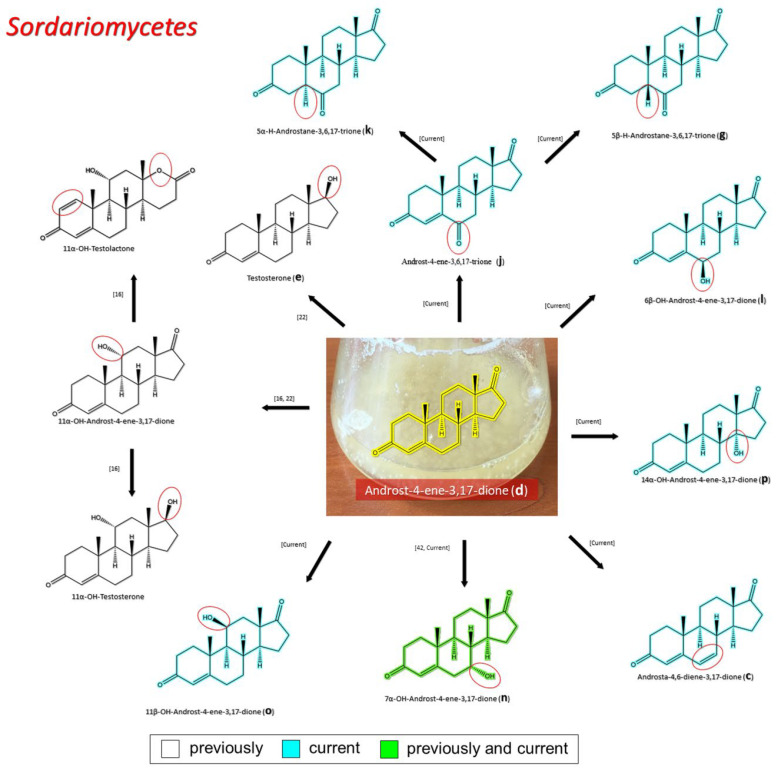
The transformation of androst-4-ene-3,17-dione (**d**) by *Sordariomycetes*. Structural formula of the substrate (**d**) is drawn in yellow and placed on the image of the flask with defined (DE) medium after 48 h of transformation for by *Simplicillium lamellicola* STG-96. Structural formulas of steroids detected during the transformation are drawn: in green for compounds found both in current and in other studies; in black for compounds found in other studies, but not found in current work; and in blue for compounds first discovered in current study. Next to the arrows are the literary sources, where modifications were described; modifications circled in red ovals.

The dominant representatives of *Sordariomycetes* among the fungi-destructors of tempera paintings from STG were strains from the *Cordycipitaceae* and *Microascaceae* families. There was only one such strain from each family; however, all of them were able to transform AD (Figure 3). Of the steroid products found in our study, 8.3% (1 out of 12) were previously described (**n**) and 58.3% (7 out of 12) of steroidal products are described for the first time (**c**, **g**, **j**, **k**, **l**, **o**, and **p**) [16,22,43] (Figure 6). If we compare the features of transformation within families of STG strains, then for *Cordycipitaceae,* 9.1% (1 out of 11) of the steroid products found in our study were previously described (**n**) and 63.6% (7 out of 11) of steroidal products are described for the first time (**c**, **g**, **j**, **k**, **l**, **o**, and **p**) (Table 2). The transformation of AD by fungi of the *Microascaceae* family is not covered in sufficient detail in the literature. There was only one study in which the only transformation product was described, TS (**e**) [22]. The STG-strain from *Microascaceae* family converted AD to four another compounds (**g**, **k**, **l**, and **p**) (Table 2). It is significant that all these four transformation products were also detected in our work for another representative of *Sordariomycetes*, the *S. lamellicola* STG-96 strain from the *Cordycipitaceae* family. Such a coincidence may indicate a relatively close metabolism of steroids in representatives of relatively close fungal taxa.

## 4. Discussion

Over the past 60–70 years, improved strains of filamentous fungi have been widely used for the needs of medical biotechnology for the production of pharmaceutically significant secondary metabolites, such as antibiotics, statins, and immunosuppressants [46,47,48,49]. On the other hand, the use of filamentous fungi for a number of stages in the transformation of steroids for medicinal purposes has become widespread in biotechnology [1,16]. In our work, we have shown for the first time that phylogenetically distant filamentous fungi belonging to the classes *Eurotiomycetes*, *Dothideomycetes*, and *Sordariomycetes*, associated by a common functional ability to grow on organic materials used in tempera painting, can transform the pharmaceutically significant steroid AD (compound **d**). On the one hand, this may be due to the ability of filamentous fungi to transform steroids as such. For example, in a recent study, approximately 73% (33 out of 45) of preliminary selected strains of *Ascomycota* and *Zygomycota* actively transformed steroids AD and ADD [22]. Moreover, ~18% (8 out of 45) of these strains did not transform AD or ADD at all the specified conditions. On the other hand, the fungi studied in our work are dominant representatives of the microbiome characterized in the main historical building of the State Tretyakov Gallery [24]. Since the middle of the 19th century, the microbiological community that exists in this museum has been constantly exposed to both conservation (including strict observance of the temperature and humidity regime) and restoration. This could lead to the selection of microorganisms for: (i) the ability to survive under pressure from conservation; (ii) the ability to use organic materials as a nutrient substrate, which are both part of the historical building itself and the exhibits [50]. In particular, one of the characteristic organic substrates found in the composition of tempera materials, and one which can potentially be used for the vital activity of destructor fungi, is cholesterol from egg yolk (a binder of tempera paints), and its derivatives that appear during the aging process of tempera paints, such as 5,6-epoxycholestan-3-ol and 3-hydroxycholest-5-en-7-one [30]. In our work, 100% (10 out of 10) of dominant fungi-destructors of tempera paints actively transformed steroid AD, which may be the primary signal for further study of such a functional group of microorganisms for the biotransformation of steroids. AD transformations under various conditions (DE medium, PPB) allowed us to more fully reveal the transformational potential (metabolic capacity) of the studied strains. Using our methods, we were unable to identify all steroid compounds obtained as a result of AD transformation by the studied group of fungi. This is due, first of all, to the fact that GC/MS allows compounds from the spectra available in databases to be determined. Despite this, we have characterized a significant number of steroid products from AD transformation (Figure 3, Figure 4 and Figure 5). Moreover, for the first time, activities not previously described in the literature were found: six previously undescribed transformation products of AD were found for the family *Aspergillaceae*, four were found for *Cladosporiaceae*, two were found for *Pleosporaceae*, seven were found for *Cordycipitaceae*, and four were found for *Microascaceae* (Table 2). In total, 33 steroids formed during the transformation of AD were characterized in the work, and for 19 of them the structure was established, which made it possible to characterize the ongoing reactions: (i) hydroxylation at positions 3α (for the compound **a**), 3β (**i**), 6β (**l**, **m**), 7α (**n**), 11β (**o**, **r**), 14α (**p**, **s**), and 15α (**q**); (ii) oxidoreductase activity at position 17 with the formation of testosterone; (iii) 5β reductase activity; (iv) C-1/C-2 dehydrogenase activity to form androstadienedione (ADD); and (v) other modifications associated with both the primary transformation of AD and subsequent modifications of the resulting intermediates. Oxidative hydroxylation of steroids (in the current study, at positions 3α, 3β, 6β, 7α, 11β, 14α, and 15α) is performed by CYP450 [15,18]. Typically, one specific enzyme of this superfamily of proteins introduces a hydroxyl group into one well-defined position of the steroid [6,51,52]. In a number of other cases, a fungal steroid hydroxylase may have more than one activity, such as the P-450_lun_ steroid 11β-/14α-hydroxylase from *Cochliobolus lunatus* [53]. In this regard, the study of steroid transformation in fungi (and other organisms) has two levels: (i) primary screening of steroid transforming activity (current study is devoted to this) [20,21,22,34]; and (ii) the establishment of the molecular basis of these activities associated with the cloning of individual genes, obtaining recombinant strains and proteins, allowing unambiguous determination of their activity [53,54,55,56].

It has also been shown that the expression of fungal CYP450 genes, the products of which are capable of transforming steroids, is induced by the addition of the transformation substrate itself [51,52,57,58]. However, a number of studies have also shown that the transformation activity of fungal strains can vary depending on the transformation conditions [20]. In our work, we used two types of transformation conditions: (i) in the liquid nutrient medium; and (ii) in PPB. As a result, it turned out that different methods of transformation for some strains led to the formation of different products and/or to different dynamics of the concentrations of common AD transformation products (Figure 3). This may be due to the activation of different mechanisms by the fungal cell; in particular, protective mechanisms against toxic concentrations of steroids, in the presence of exogenous nutrient resources (during transformation in a nutrient medium) or in their absence (transformation into a buffer) [59].

A characteristic feature of the studied group of filamentous fungi was the appearance after 10–15 days of characteristic pigment colors associated with the biosynthesis of secondary metabolites (Figure 1) [25,27,60]. The biosynthesis of secondary metabolites also indicates a switch in fungal development from the tropophase to the idiophase stage [61]. It is known that steroid compounds are also synthesized among secondary metabolites [13,62]. In this regard, one of the further directions for studying the biotransformation potential of fungi-destructors of tempera paintings is their long-term cultivation with steroid substrates in order to study for the appearance of additional steroid-transforming reactions during the idiophase.

This study is important because it opens the way for future research: (i) the demonstrated fundamental ability to transform AD with the studied fungi allows us to continue the search for biotransformation activities in relation to other pharmaceutically important steroids in order to optimize conditions and scale for industrial use; and (ii) representatives of a fairly large group of fungi-destructors of tempera paintings are generally seen as promising objects for screening the biotransformation of steroids.

## 5. Conclusions

In this work, it was shown for the first time that fungi-destructors of tempera paintings show a steroid-transforming activity and are promising microorganisms for the screening of biotechnologically significant transformations of steroids with further industrial use.

## Figures and Tables

**Table 1:** GC/MS values and structures of steroid substrate and bioconversion products formed with filamentous fungi-destructors of tempera painting materials.

Compound	R_T_ (min)	MW	Structure	Encoding
3α-OH-5β-H-Androstane-17-one	11.23	290	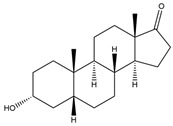	**a**
5β-H-Androstane-3,17-dione	11.50	288	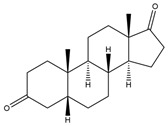	**b**
Androsta-4,6-diene-3,17-dione (6-Dehydroandrostenedione)	12.45	284	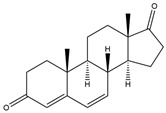	**c**
Androst-4-ene-3,17-dione (AD, Androstenedione)	12.51	286	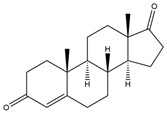	**d**
17β-Hydroxyandrost-4-en-3-one (TS, Testosterone)	12.64	288	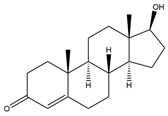	**e**
Androsta-1,4-diene-3,17-dione (ADD, Androstadienedione, Boldione)	12.73	284	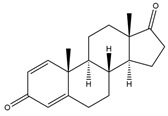	**f**
5β-H-Androstane-3,6,17-trione	12.88	302	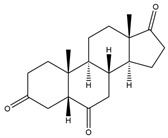	**g**
Androst-4-ene-3,11,17-trione (Androstane-3,11,17-trione)	13.11	300	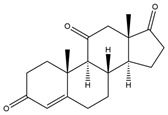	**h**
3β-OH-5α-H-Androstane-6,17-dione	13.42	304	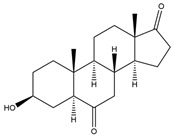	**i**
Androst-4-ene-3,6,17-trione	13.69	300	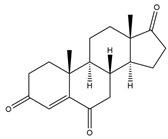	**j**
5α-H-Androstane-3,6,17-trione	13.79	302	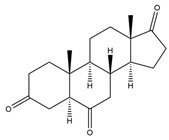	**k**
6β-OH-Androst-4-ene-3,17-dione (6β-OH-AD)	13.92	302	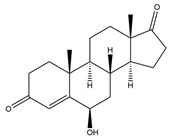	**l**
6β-OH-Testosterone (6β-OH-TS)	14.28	304	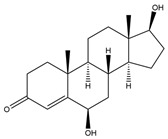	**m**
7α-OH-Androst-4-ene-3,17-dione (7α-OH-AD)	14.60	302	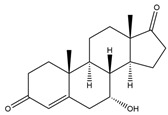	**n**
11β-OH-Androst-4-ene-3,17-dione (11β-OH-AD)	14.74	302	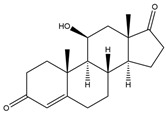	**o**
14α-OH-Androst-4-ene-3,17-dione (14α-OH-AD)	14.83	302	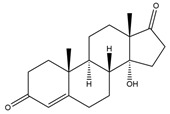	**p**
15α-OH-Androst-4-ene-3,17-dione (15α-OH-AD)	14.90	302	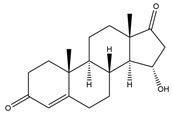	**q**
11β-OH-Testosterone (11β-OH-TC)	15.33	304	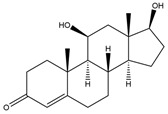	**r**
14α-OH-Androsta-1,4-diene-3,17-dione (14α-OH-ADD)	15.42	300	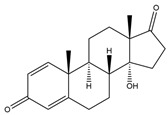	**s**

**Table 2:** Steroids formed as a result of AD (compound **b**) transformation by filamentous fungi.

Filamentous Fungi		The Product of Transformation	Source
Class	Family		
*Eurotiomycetes*	*Aspergillaceae*	Androst-4,6-diene-3,17-dione (**c**) TS (**e**) ^1^ ADD (**f**) 5β–H–Androstane-3,6,17-trione (**g**) Androstane-3,11,17-trione (**h**) 5α–H–Androstane-3,6,17-trione (**k**) 6β–OH–AD (**l**) 6β–OH–TS (**m**) 11β–OH–AD (**o**) 14α–OH–AD (**p**) 14α–OH–ADD (**s**)	Current
		TS (**e**) Testolactone	[33]
		15α–OH–AD (**q**) 17α-Oxa-D-homo-5α-androstan-3,17-dione Testolactone	[16]
		7β–OH–AD 11α–OH–AD	[34]
		1α–OH–AD 1α,6β–dihydroxy–AD	[35]
		Testololactone	[36,37]
		6β–OH–TS (**m**) 14α–OH–TS 15α–OH–TS	[38]
		11α-OH-AD	[39]
		TS (**e**) 6β–OH–AD (**l**) 11α–OH–AD 11α–OH–TS 11β –OH–AD (**o**) 14α–OH–AD (**p**) 15α–OH–TS 15β –OH–TS Testololactone	[40]
*Dothideomycetes*	*Cladosporiaceae*	TS (**e**) ADD (**f**) 5β–H–Androstane-3,6,17-trione (**g**) 3β–OH–5α–H–Androstane-6,17-dione (**i**) 5α–H–Androstane-3,6,17-trione (**k**) 6β–OH–AD (**l**) 6β–OH–TS (**m**) 15α–OH–AD (**q**)	Current
		TS (**e**) 5α–H–Androstane-3,6,17-trione (**k**) 6β–OH–AD (**l**) 17β–OH–androst-4-en-3,16-dione 15α–OH-AD (**q**) 6β,17β dihydroxyandrost-4-en-3,16-dione	[23]
		TS (**e**) 17β-Hydroxyandrost-4-ene-3,16-dione 16β,17β-Dihydroxyandrost-4-ene-3-one 16β,17α-Dihydroxyandrost-4-ene-3-one	[41]
	*Pleosporaceae* (except genus *Curvularia*)	3α–OH–5β–H–Androstane-17-one (**a**) 5β–H–Androstane-6,17-dione (**b**) TS (**e**) 14α–OH–AD (**p**)	Current
		TS (**e**) 7α–OH–AD (**n**) 7β–OH–AD 7α–OH–TS 14α–OH–AD (**p**)	[22]
		5α–H–Androstane-3,6,17-trione (**k**) 7α–OH–AD (**n**) 7β–OH–AD 14α–OH–AD (**p**) 17β-Hydroxy-5α-Androstane-3,6-dione	[23]
	*Pleosporaceae* (genus *Curvularia*)	6β–OH–AD (**l**) 6β–OH–TS (**m**) 11β-OH AD (**o**) 14α–OH–AD (**p**) 11β–OH–TS (**r**)	Current
		TS (**e**) 7α-OH-TS 11β-OH AD (**o**) 14α-OH-AD (**p**) 11β-OH-TS (**r**) 14α-OH-TS	[20]
		11β-OH AD (**o**) 14α-OH-AD (**p**)	[32]
		11α–OH–AD	[42]
*Sordariomycetes*	*Cordycipitaceae*	Androst-4,6-diene-3,17-dione (**c**) 5β–H–Androstane-3,6,17-trione (**g**) Androst-4-ene-3,6,17-trione (**j**) 5α–H–Androstane-3,6,17-trione (**k**) 6β–OH–AD (**l**) 7α–OH–AD (**n**) 11β-OH-AD (**o**) 14α–OH–AD (**p**)	Current
		11α–OH–AD 11α–OH–TS 11α-OH-testolactone	[16]
		7α–OH–AD (**n**)	[43]
		11α–OH–AD	[22]
	*Microascaceae*	5β–H–Androstane-3,6,17-trione (**g**) 5α–H–Androstane-3,6,17-trione (**k**) 6β–OH–AD (**l**) 14α–OH–AD (**p**)	Current
		TS (**e**)	[22]

^1^ Underlined letters indicate compounds described both in the current work and in the literature for fungi within the same family (for *Pleosporaceae*—either without and only with the genus *Curvularia*).

## Data Availability

The data presented in this study are contained within the article.

## Supplementary Materials

The following supporting information can be downloaded at: https://www.mdpi.com/2079-7737/11/6/883/s1, Figure S1: GC/MS data for STG-36 strain, 0 day (start point) of Androst-4-ene-3,17-dione (**d**) transformation on DE medium. The formula of the steroid substrate **d** is filled in red; Figure S2: GC/MS data for STG-36 strain, 2 days of Androst-4-ene-3,17-dione (**d**) transformation on DE medium. Steroid formulas are filled in: in red for the substrate (**d**), in green for steroid transformation products **a** (3α-OH-5β-H-Androstane-17-one) and **b** (5β-H-Androstane-3,17-dione); Figure S3: GC/MS data for STG-57 strain, 3 days of Androst-4-ene-3,17-dione (**d**) transformation on DE medium. Steroid formulas are filled in: in red for the substrate (**d**), in green for steroid transformation products **g** (5β-H-Androstane-3,6,17-trione), **k** (5α-H-Androstane-3,6,17-trione), **l** (6β-OH-Androst-4-ene-3,17-dione), and **p** (14α-OH-Androst-4-ene-3,17-dione); Figure S4: GC/MS data for STG-96 strain, 2 days of Androst-4-ene-3,17-dione (**d**) transformation on DE medium. Steroid formulas are filled in: in red for the substrate (**d**), in green for steroid transformation products **c** (Androsta-4,6-diene-3,17-dione), **g** (5β-H-Androstane-3,6,17-trione), **k** (5α-H-Androstane-3,6,17-trione), **l** (6β-OH-Androst-4-ene-3,17-dione), **o** (11β-OH-Androst-4-ene-3,17-dione), and **p** (14α-OH-Androst-4-ene-3,17-dione); Figure S5: GC/MS data for STG-93B strain, 2 days of Androst-4-ene-3,17-dione (**d**) transformation on PPB. Steroid formulas are filled in: in red for the substrate (**d**), in green for steroid transformation products **g** (5β-H-Androstane-3,6,17-trione) and **k** (5α-H-Androstane-3,6,17-trione); Figure S6: Thin layer chromatography data for STG-25G and STG-86 strains after 2 and 3 days of Androst-4-ene-3,17-dione (**d**) transformation on DE medium. M—mixture of steroid standards.

## Abbreviations

AD	Androstenedione (Androst-4-ene-3,17-dione)
ADD	Androstadienedione (Androsta-1,4-diene-3,17-dione)
CDA	Czapek-Dox agar
CYP450	Cytochromes P450
GC/MS	Gas chromatography/mass spectrometry
DE	Defined (medium)
PPB	Potassium phosphate buffer
SE	Seed (medium)
STG	State Tretyakov Gallery (museum, Moscow, Russia)
TLC	Thin layer chromatography
TM1	Transformation method 1
TM2	Transformation method 2
TS	Testosterone (17β-Hydroxyandrost-4-en-3-one)

## References

[1] Fernández-Cabezón L., Galán B., García J.L. (2018). New insights on steroid biotechnology. Front. Microbiol..

[2] Cano-Flores A., Gómez J., Ramos R. (2020). Biotransformation of Steroids Using Different Microorganisms. Chemistry and Biological Activity of Steroids.

[3] Torgov I.V. (1963). Progress in the total synthesis of steroids. Pure Appl. Chem..

[4] Carballeira J.D., Quezada M.A., Hoyos P., Simeó Y., Hernaiz M.J., Alcantara A.R., Sinisterra J.V. (2009). Microbial cells as catalysts for stereoselective red-ox reactions. Biotechnol. Adv..

[5] Fernandes P., Cruz A., Angelova B., Pinheiro H.M., Cabral J.M.S. (2003). Microbial conversion of steroid compounds: Recent developments. Enzym. Microb. Technol..

[6] Lu W., Feng J., Chen X., Bao Y.J., Wang Y., Wu Q., Ma Y., Zhu D. (2019). Distinct regioselectivity of fungal P450 enzymes for steroidal hydroxylation. Appl. Environ. Microbiol..

[7] Bensasson C.S., Hanson J.R., Le Huerou Y. (1999). The microbiological hydroxylation of 3α,5-cycloandrostanes by *Cephalosporium aphidicola*. Phytochemistry.

[8] Donova M.V. (2007). Transformation of steroids by actinobacteria: A review. Appl. Biochem. Microbiol..

[9] Jaderets V.V., Andrjushina V.A., Vojshvillo N.E., Dvojnikov P.S., Druzhinina A.V., Stytsenko T.S., Zejnalov O.A., Skrjabin K.G. (2010). Method for Preparation 14α-Hydroxyderivatives of Δ4-3,17-Diketo-Androstene. Patent.

[10] Zhgun A.A., Soloneski S., Larramendy M.L. (2021). Random Mutagenesis of Filamentous Fungi Stains for High-Yield Production of Secondary Metabolites: The Role of Polyamines. Genotoxicity and Mutagenicity—Mechanisms and Test Methods, Chapter 2.

[11] Avalos J., Limón M.C. (2022). Fungal Secondary Metabolism. Encyclopedia.

[12] Keller N.P. (2019). Fungal secondary metabolism: Regulation, function and drug discovery. Nat. Rev. Microbiol..

[13] Ortega H.E., Torres-Mendoza D., Caballero E.Z., Cubilla-Rios L. (2021). Structurally Uncommon Secondary Metabolites Derived from Endophytic Fungi. J. Fungi.

[14] Zheng R., Li S., Zhang X., Zhao C. (2021). Biological Activities of Some New Secondary Metabolites Isolated from Endophytic Fungi: A Review Study. Int. J. Mol. Sci..

[15] Kristan K., Rižner T.L. (2012). Steroid-transforming enzymes in fungi. J. Steroid Biochem. Mol. Biol..

[16] Nassiri-Koopaei N., Faramarzi M.A. (2015). Recent developments in the fungal transformation of steroids. Biocatal. Biotransform..

[17] Park J., Lee S., Choi J., Ahn K., Park B., Park J., Kang S., Lee Y.H. (2008). Fungal cytochrome P450 database. BMC Genom..

[18] Durairaj P., Hur J.S., Yun H. (2016). Versatile biocatalysis of fungal cytochrome P450 monooxygenases. Microb. Cell Fact..

[19] Hüttel W., Hoffmeister D. (2011). Fungal Biotransformations in Pharmaceutical Sciences. Industrial Applications.

[20] Karpova N.V., Andryushina V.A., Stytsenko T.S., Druzhinina A.V., Feofanova T.D., Kurakov A.V. (2016). A search for microscopic fungi with directed hydroxylase activity for the synthesis of steroid drugs. Appl. Biochem. Microbiol..

[21] Kollerov V.V., Shutov A.A., Kazantsev A.V., Donova M.V. (2019). Biocatalytic modifications of pregnenolone by selected filamentous fungi. Biocatal. Biotransform..

[22] Kollerov V., Shutov A., Kazantsev A., Donova M. (2020). Biotransformation of androstenedione and androstadienedione by selected *Ascomycota* and *Zygomycota* fungal strains. Phytochemistry.

[23] Yildirim K., Kuru A., Küçükbaşol E. (2020). Microbial transformation of androstenedione by *Cladosporium sphaerospermum* and *Ulocladium chartarum*. Biocatal. Biotransform..

[24] Zhgun A., Avdanina D., Shumikhin K., Simonenko N., Lyubavskaya E., Volkov I., Ivanov V. (2020). Detection of potential biodeterioration risks for tempera painting in 16th century exhibits from State Tretyakov Gallery. PLoS ONE.

[25] Alexandrova L.A., Shevchenko O.V., Jasko M.V., Solyev P.N., Karpenko I.L., Negrya S.D., Efremenkova O.V., Vasilieva B.F., Efimenko T.A., Avdanina D.A. (2022). 3′-Amino modifications enhance the antifungal properties of N4-alkyl-5-methylcytidines for potential biocides. New J. Chem..

[26] Alexandrova L.A., Jasko M.V., Negrya S.D., Solyev P.N., Shevchenko O.V., Solodinin A.P., Kolonitskaya D.P., Karpenko I.L., Efremenkova O.V., Glukhova A.A. (2021). Discovery of novel N^4^-alkylcytidines as promising antimicrobial agents. Eur. J. Med. Chem..

[27] Zhgun A.A., Avdanina D.A., Shagdarova B.T., Troyan E.V., Nuraeva G.K., Potapov M.P., Il’ina A.V., Shitov M.V., Varlamov V.P. (2020). Search for Efficient Chitosan-Based Fungicides to Protect the 15th–16th Centuries Tempera Painting in Exhibits from the State Tretyakov Gallery. Microbiology.

[28] Masschelein-Kleiner L., Lawrence T. (1995). Ancient Binding Media, Varnishes and Adhesives.

[29] Stadelman W.J., Cotterill O.J. (1995). Egg Science and Technology.

[30] van den Brink O.F., Ferreira E.S.B., van der Horst J., Boon J.J. (2009). A direct temperature-resolved tandem mass spectrometry study of cholesterol oxidation products in light-aged egg tempera paints with examples from works of art. Int. J. Mass Spectrom..

[31] Wallace W.E., Ji W., Tchekhovskoi D.V., Phinney K.W., Stein S.E. (2017). Mass Spectral Library Quality Assurance by Inter-Library Comparison. J. Am. Soc. Mass Spectrom..

[32] Andryushina V.A., Voishvillo N.E., Druzhinina A.V., Stytsenko T.S., Yaderets V.V., Petrosyan M.A., Zeinalov O.A. (2013). 14α-Hydroxylation of steroids by mycelium of the mold fungus Curvularia lunata (VKPM F-981) to produce precursors for synthesizing new steroidal drugs. Pharm. Chem. J..

[33] Faramarzi M.A., Yazdi M.T., Amini M., Mohseni F.A., Zarrini G., Amani A., Shafiee A. (2004). Microbial production of testosterone and testololactone in the culture of *Aspergillus terreus*. World J. Microbiol. Biotechnol..

[34] Heidary M., Ghasemi S., Habibi Z., Ansari F. (2020). Biotransformation of androst-4-ene-3,17-dione and nandrolone decanoate by genera of *Aspergillus* and *Fusarium*. Biotechnol. Lett..

[35] Mao S., Zhang L., Ge Z., Wang X., Li Y., Liu X., Liu F., Lu F. (2016). Microbial hydroxylation of steroids by *Penicillium decumbens*. J. Mol. Catal. B Enzym..

[36] Panek A., Łyczko P., Świzdor A. (2020). Microbial Modifications of Androstane and Androstene Steroids by *Penicillium vinaceum*. Molecules.

[37] Kołek T., Szpineter A., Świzdor A. (2008). Baeyer-Villiger oxidation of DHEA, pregnenolone, and androstenedione by *Penicillium lilacinum* AM111. Steroids.

[38] Yildirim K., Kuru A. (2016). The Biotransformation of Some Steroids by *Aspergillus Sydowii* MRC 200653. J. Chem. Res..

[39] Ríos L.O.D.L., Luengo J.M., Fernández-Cañón J.M. (2017). Steroid 11-alpha-hydroxylation by the fungi *Aspergillus nidulans* and *Aspergillus ochraceus*. Methods Mol. Biol..

[40] Yildirim K., Kuru A., Keskin E., Salihoglu A., Bukum N. (2017). Biotransformation of Androst-4-Ene-3,17-Dione by Some Fungi. J. Chem. Res..

[41] Yildirim K., Kuru A., Yılmaz R.F. (2018). Microbial Transformation of Some Steroids by *Cladosporium Cladosporioides* Mrc 70282. J. Chem. Res..

[42] Choudhary M.I., Sultan S., Khan M.T.H., Yasin A., Shaheen F., Atta-Ur-Rahman A. (2007). Biotransformation of (+)-androst-4-ene-3,17-dione. Nat. Prod. Res..

[43] Kozłowska E., Dymarska M., Kostrzewa-Susłow E., Janeczko T. (2017). Isaria fumosorosea KCh J2 Entomopathogenic Strain as an Effective Biocatalyst for Steroid Compound Transformations. Molecules.

[44] Črešnar B., Petrič Š. (2011). Cytochrome P450 enzymes in the fungal kingdom. Biochim. Biophys. Acta.

[45] Karunarathna S.C., Damodara Shenoy B., Pripdeevech P., Madawala S., Tang A.M.C., Karbowy-Thongbai B., Dissanayake A.J., Dutta A.K., Palnam Dauda W., Abraham P. (2022). Robust Profiling of Cytochrome P450s (P450ome) in Notable *Aspergillus* spp.. Life.

[46] Fierro F., Vaca I., Castillo N.I., García-Rico R.O., Chávez R. (2022). *Penicillium chrysogenum*, a Vintage Model with a Cutting-Edge Profile in Biotechnology. Microorganisms.

[47] Zhgun A.A., Eldarov M.A. (2021). Polyamines Upregulate Cephalosporin C Production and Expression of β-Lactam Biosynthetic Genes in High-Yielding *Acremonium chrysogenum* Strain. Molecules.

[48] Zhgun A.A., Dumina M.V., Voinova T.M., Dzhavakhiya V.V., Eldarov M.A. (2018). Role of acetyl-CoA Synthetase and LovE Regulator Protein of Polyketide Biosynthesis in Lovastatin Production by Wild-Type and Overproducing *Aspergillus terreus* Strains. Appl. Biochem. Microbiol..

[49] Domratcheva A.G., Zhgun A.A., Novak N.V., Dzhavakhiya V.V. (2018). The Influence of Chemical Mutagenesis on the Properties of the Cyclosporine a High-Producer Strain *Tolypocladium inflatum* VKM F-3630D. Appl. Biochem. Microbiol..

[50] Pinheiro A.C., Sequeira S.O., Macedo M.F. (2019). Fungi in archives, libraries, and museums: A review on paper conservation and human health. Crit. Rev. Microbiol..

[51] Felpeto-Santero C., Galán B., Luengo J.M., Fernández-Cañon J.M., Del Cerro C., Medrano F.J., García J.L. (2019). Identification and expression of the 11β-steroid hydroxylase from *Cochliobolus lunatus* in *Corynebacterium glutamicum*. Microb. Biotechnol..

[52] Petrič Š., Hakki T., Bernhardt R., Žigon D., Črešnar B. (2010). Discovery of a steroid 11α-hydroxylase from *Rhizopus oryzae* and its biotechnological application. J. Biotechnol..

[53] Chen J., Tang J., Xi Y., Dai Z., Bi C., Chen X., Fan F., Zhang X. (2019). Production of 14α-hydroxysteroids by a recombinant *Saccharomyces cerevisiae* biocatalyst expressing of a fungal steroid 14α-hydroxylation system. Appl. Microbiol. Biotechnol..

[54] Felpeto-Santero C., Galán B., García J.L. (2021). Engineering the steroid hydroxylating system from *Cochliobolus lunatus* in *Mycolicibacterium smegmatis*. Microorganisms.

[55] Hull C.M., Warrilow A.G.S., Rolley N.J., Price C.L., Donnison I.S., Kelly D.E., Kelly S.L. (2017). Co-production of 11α-hydroxyprogesterone and ethanol using recombinant yeast expressing fungal steroid hydroxylases. Biotechnol. Biofuels.

[56] Jgoun A.A., Eldarov M.A., Solodar L.I., Sokolov N.N., Archakov A.I., Skryabin K.G. (2001). Heterologous expression of eukaryotic CYP450. 1. Heterologous expression of cytochrome P450 2B4 using groups with various affinity in *E. coli*. Vopr. Med. Khim..

[57] Breskvar K., Hudnik-Plevnik T. (1981). Inducibility of cytochrome P-450 and of NADPH-cytochrome C reductase in progesterone treated filamenteous fungi *Rhizopus nigricans* and *Rhizopus arrhizus*. J. Steroid Biochem..

[58] Irrgang S., Schlosser D., Schmauder H.P. (1992). The steroid 15α-hydroxylase of *Penicillium raistrickii* I 477 is inducible. Biotechnol. Lett..

[59] Črešnar B., Žakelj-Mavrič M. (2009). Aspects of the steroid response in fungi. Chem. Biol. Interact..

[60] Hyvönen M.T., Keinänen T.A., Nuraeva G.K., Yanvarev D.V., Khomutov M., Khurs E.N., Kochetkov S.N., Vepsäläinen J., Zhgun A.A., Khomutov A.R. (2020). Hydroxylamine analogue of agmatine: Magic bullet for arginine decarboxylase. Biomolecules.

[61] Demain A.L. (1986). Regulation of secondary metabolism in fungi. Pure Appl. Chem..

[62] Sultan A. (2015). Steroids: A Diverse Class of Secondary Metabolites. Med. Chem..

