# Supplementary figures and images for: Biotransformation of Androstenedione by Filamentous Fungi Isolated from Cultural Heritage Sites in the State Tretyakov Gallery
Source: Biology (Basel). 2022 Jun 8;11(6):883. doi: 10.3390/biology11060883 (PMC9220046; doi:10.3390/biology11060883)

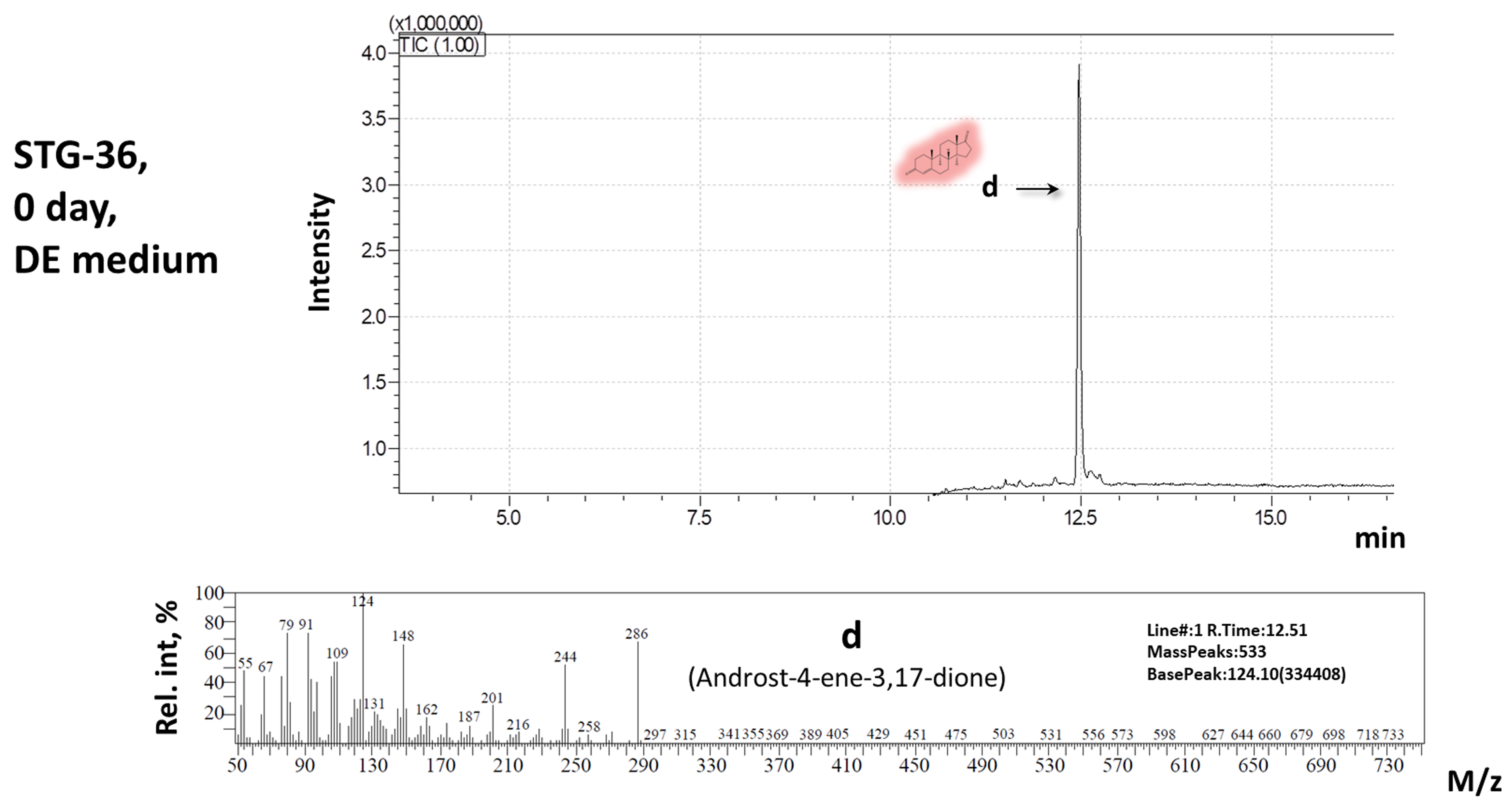

Supplement: Supplementary file 1 [file biology-11-00883-s001.zip › Figure S1.tif]

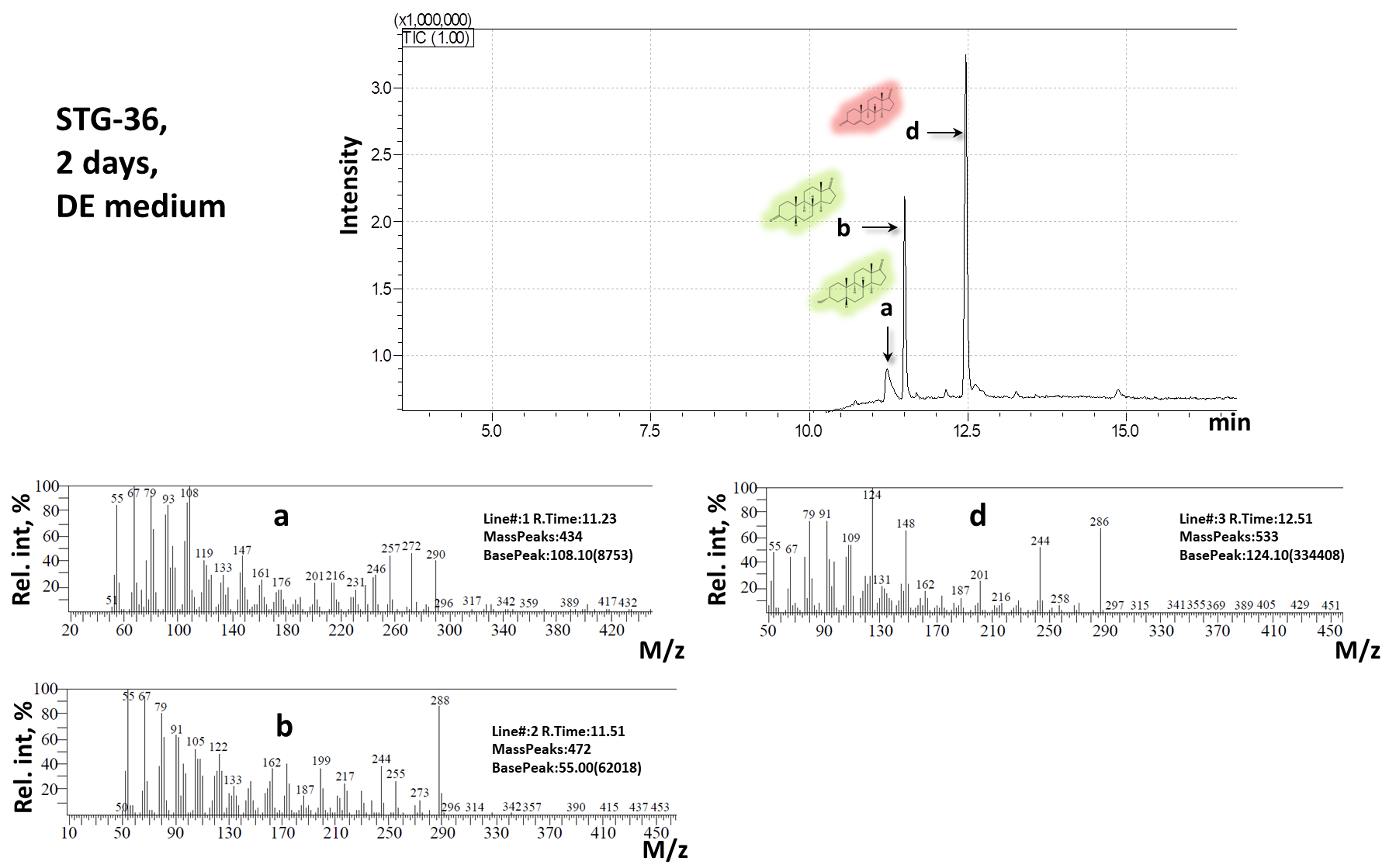

Supplement: Supplementary file 1 [file biology-11-00883-s001.zip › Figure S2.tif]

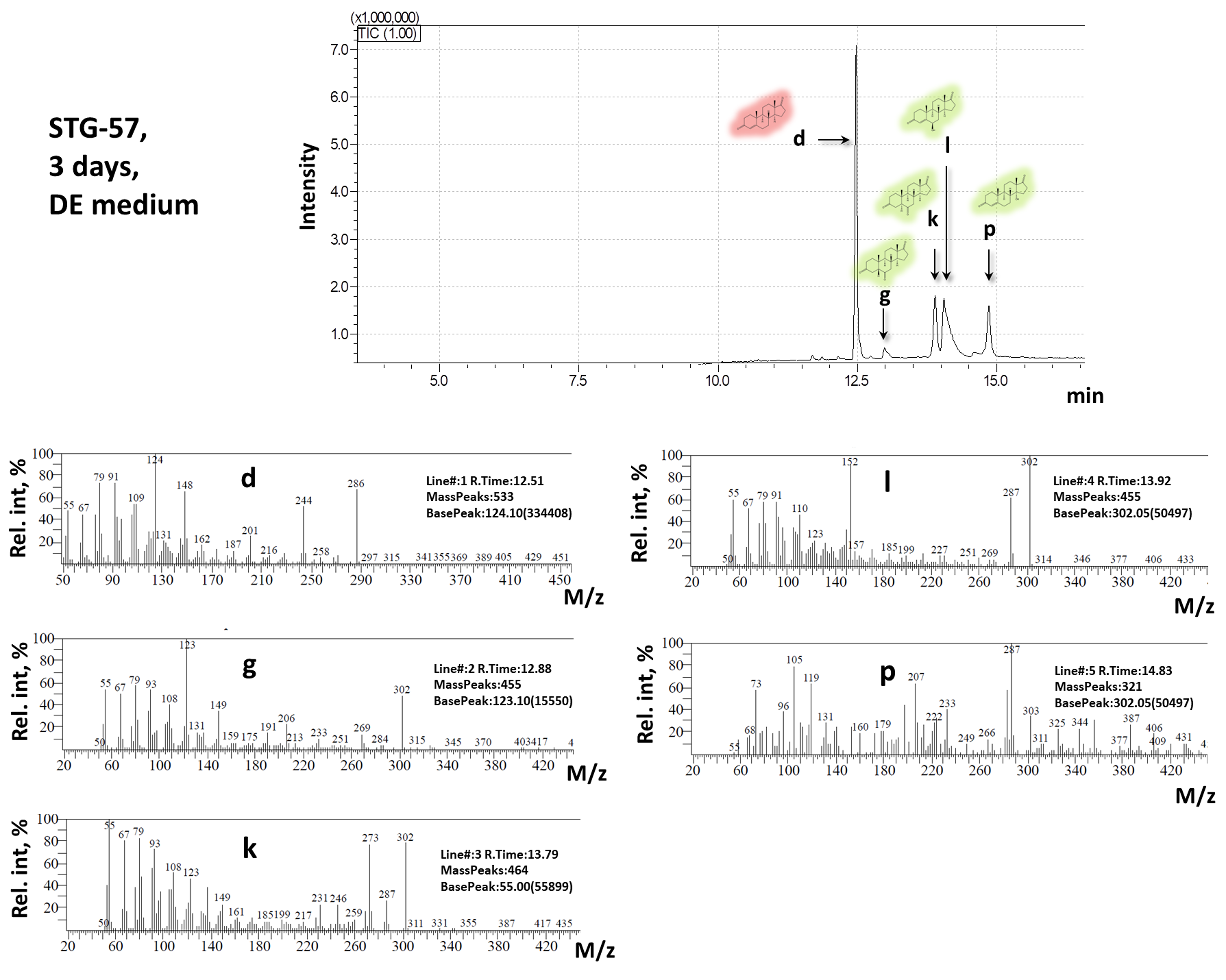

Supplement: Supplementary file 1 [file biology-11-00883-s001.zip › Figure S3.tif]

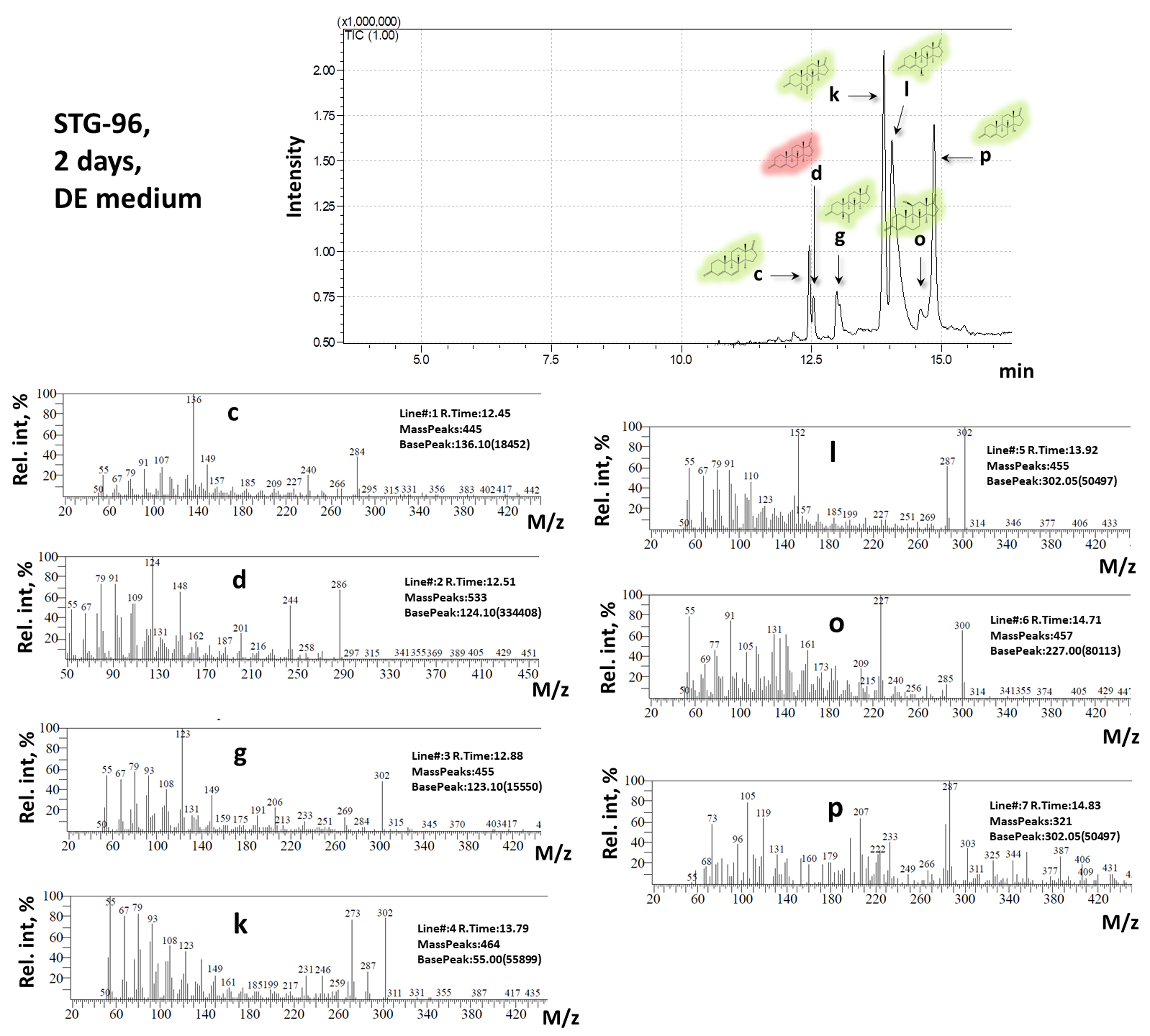

Supplement: Supplementary file 1 [file biology-11-00883-s001.zip › Figure S4.tif]

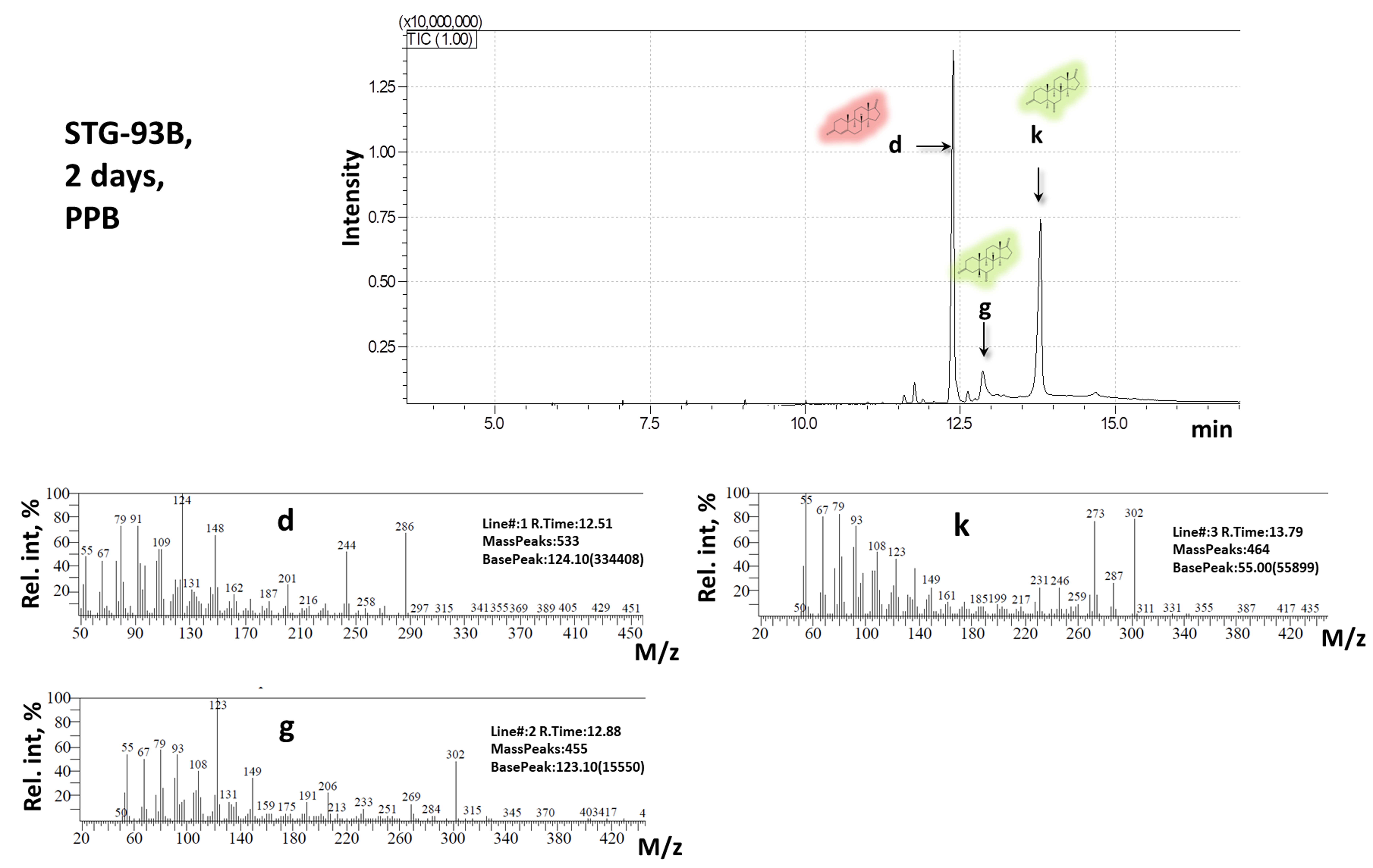

Supplement: Supplementary file 1 [file biology-11-00883-s001.zip › Figure S5.tif]

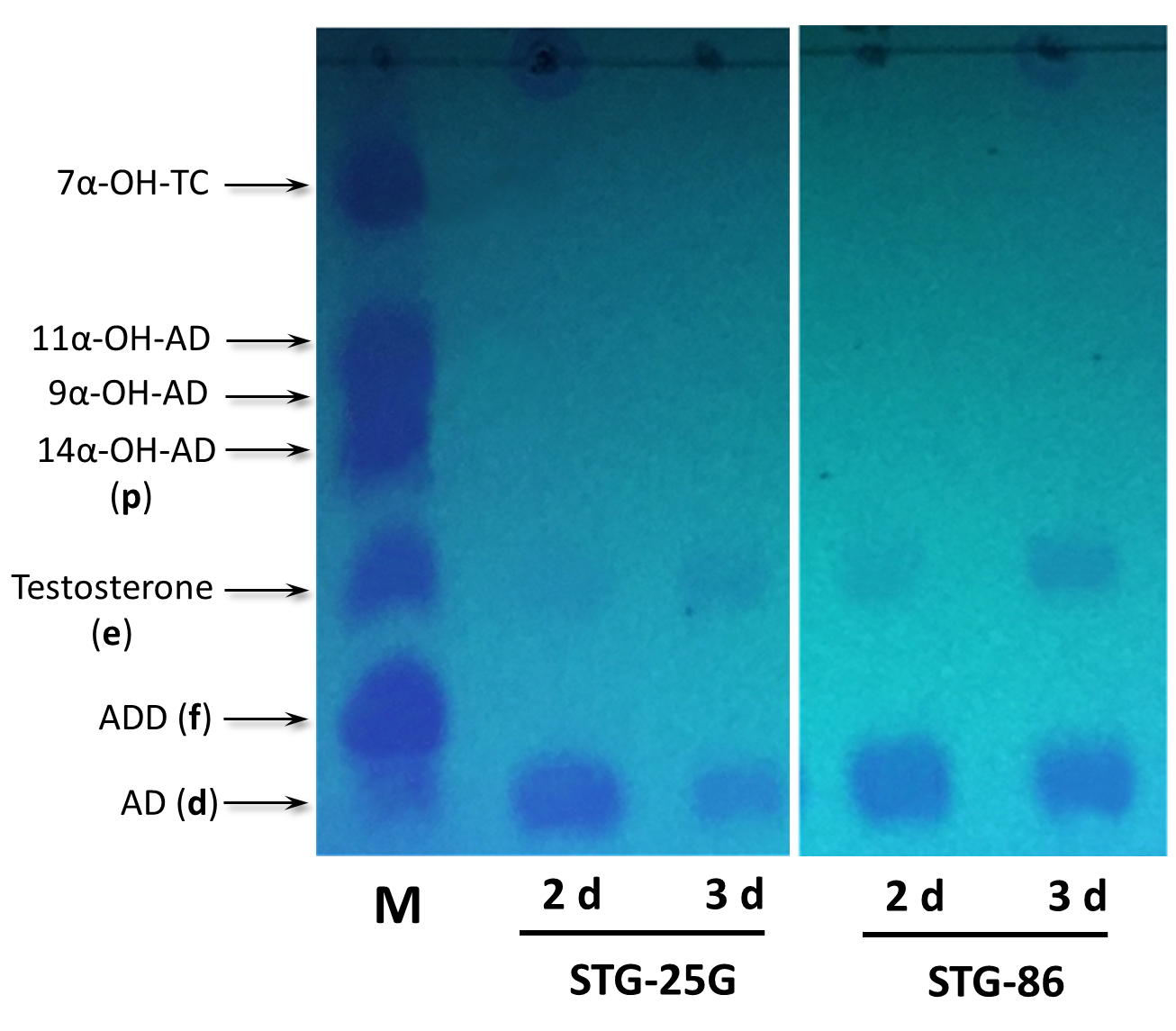

Supplement: Supplementary file 1 [file biology-11-00883-s001.zip › Figure S6.tif]
